# Retraction: 3, 3′-diindolylmethane Enhances the Effectiveness of Herceptin against HER-2/Neu-Expressing Breast Cancer Cells

**DOI:** 10.1371/journal.pone.0346218

**Published:** 2026-04-08

**Authors:** The PLOS One Editors

The *PLOS One* Editors retract this article [1] due to concerns about reliance on retracted work cited in References 17, 18, 32, 33, 35, 36, and 42.

In addition, the following panels appear similar despite representing different experimental conditions:

- The SKBR3 PARP panel in Fig 2B of [1] and the PARP panel in Fig 1C of [2, retracted in 3].
- The MDA-MB-468 PARP panel in Fig 2B of [1] and the Caspase-9 panel in Fig 1C of [2, retracted in 3].
- The FoxM1 and pAKT panels in Fig 4F of [1] and lanes 1-3 of the Cdk6 and Cdk4 panels in Fig 5A of [4].
- The β-actin panel in Fig 4F of [1] and lanes 2-4 of the Actin panel in Fig 5B of [4].

All authors either did not respond directly or could not be reached.

The PARP panels in Fig 2B of [1] report material similar to that published in [2, retracted in 3], published in 2009 by the American Association for Cancer Research, which are not offered under a CC BY license and are therefore excluded from this article’s [1] license.

The FoxM1, pAKT, and β-actin panels in Fig 4F of [1] appear similar to content previously published in [4] under a CC BY 2.0 license. These panels are subject to the license that applies to [4].
